# Crispy Cravings May Affect Baby’s Health: Prenatal Acrylamide Exposure Is Associated with Reduced Birth Weight

**DOI:** 10.1289/ehp.120-a475b

**Published:** 2012-12-03

**Authors:** Julia R. Barrett

**Affiliations:** Julia R. Barrett, MS, ELS, a Madison, WI–based science writer and editor, has written for *EHP* since 1996. She is a member of the National Association of Science Writers and the Board of Editors in the Life Sciences.

Many commonly eaten foods contain acrylamide, a known neurotoxicant and suspected human carcinogen. Previous animal studies have suggested acrylamide may also cause reduced birth weight in prenatally exposed offspring. Investigators now report an association between higher concentrations of acrylamide and glycidamide hemoglobin adducts in umbilical cord blood and reduced birth weight in children of women who ate diets rich in acrylamide [*EHP* 120(12):1739–1745; Pedersen et al.].

Acrylamide forms during frying or high-temperature baking of carbohydrate-rich foods such as fried potatoes, potato chips, cookies, and breakfast cereals. Within the body, acrylamide and its metabolite glycidamide form adducts, or chemically joined products, with proteins and DNA. These adducts can potentially disrupt genetic and biological functions.

Pregnant women at 11 locations in Denmark, Greece, Norway, Spain, and England participated in the study during 2006–2011, and the investigators used information on 1,101 mother–child pairs. Demographic and detailed dietary information was collected from all the women, umbilical cord blood samples were obtained at birth, and birth information was extracted from medical records.

Acrylamide and glycidamide hemoglobin adducts were found in all cord blood samples, and levels tended to be higher if the mother smoked. Subsequent analyses accounted for maternal smoking as well as other potentially confounding factors, such as length of pregnancy, child’s sex and country of birth, and mother’s age, prepregnancy weight, and ethnicity.

**Figure:**
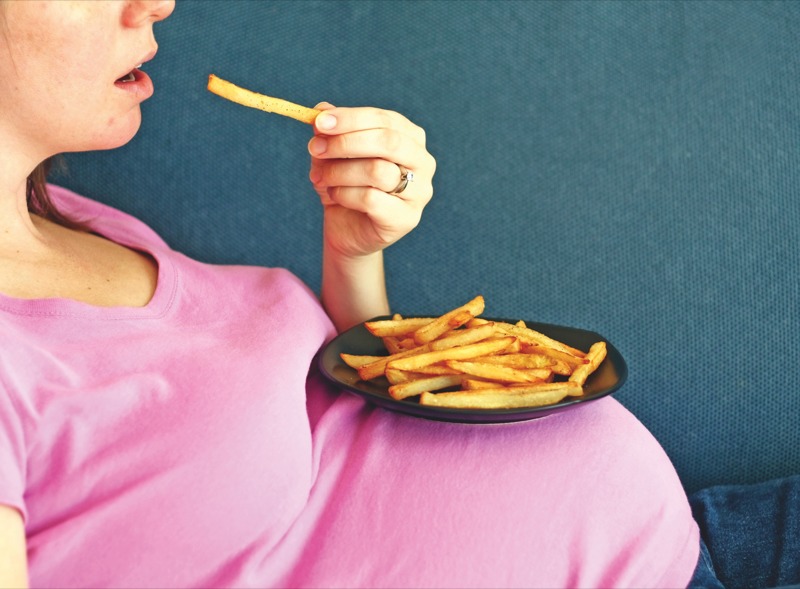
New findings suggest that eating a diet rich in acrylamide during pregnancy could increase the risk of low birth weight. © 2012 Rob Mattingley/iStockphoto.com

The investigators found an association between decreasing birth weight and increasing levels of adducts. In a comparison of the highest and lowest quartiles of exposure among children of nonsmoking mothers, acrylamide and glycidamide were associated with average birth weight decreases of 107 and 103 g (3.8 and 3.6 oz), respectively. These decreases are comparable with those attributed to moderate smoking during pregnancy. Reduced birth weight is linked with adverse health effects such as impaired growth and development early in life and increased incidence of cardiovascular disease, diabetes, and osteoporosis in adulthood.

The availability of adduct measurements and the large, well-characterized study population are key strengths of the study. However, there remained the potential for unmeasured or uncontrolled factors to influence the results. Furthermore, it was not possible to precisely characterize dietary acrylamide intake, because even in the same types of foods, levels can vary depending on manufacturing and preparation methods. If confirmed by further research, the results of this study suggest that pregnant women may benefit from reduced consumption of acrylamide-containing foods.

